# The Acute Radiation Syndrome-Mitigator Romiplostim and Secreted Extracellular Vesicles Improved Survival in Mice Acutely Exposed to Myelosuppressive Doses of Ionizing Radiation

**DOI:** 10.3390/biom13050837

**Published:** 2023-05-15

**Authors:** Masaru Yamaguchi, Ikuo Kashiwakura

**Affiliations:** Graduate School of Health Sciences, Hirosaki University, 66-1 Hon-cho, Hirosaki 036-8564, Aomori, Japan; masarun@hirosaki-u.ac.jp

**Keywords:** romiplostim, extracellular vesicles, hematopoietic acute radiation syndrome, radiological or nuclear public health emergency, microRNA

## Abstract

In cases of accidental high-dose total-body irradiation (TBI), acute radiation syndrome (ARS) can cause death. We reported that the thrombopoietin receptor agonist romiplostim (RP) has the potential to completely rescue mice exposed to lethal TBI. Extracellular vesicles (EVs) are involved in cell-to-cell communication, and the mechanism of RP action may be related to EVs that reflect the radio-mitigative information. We investigated the radio-mitigative effects of EVs on mice with severe ARS. C57BL/6 mice exposed to lethal TBI were treated with RP, and the EVs were isolated from the serum and intraperitoneally injected into other mice with severe ARS. The 30-day survival rate of lethal TBI mice drastically improved by 50–100% with the administration of EVs in the sera collected weekly from the mice in which radiation damage was alleviated and mortality was avoided by the administration of RP. Four responsive miRNAs, namely, miR-144-5p, miR-3620-5p, miR-6354, and miR-7686-5p showed significant expression changes in an array analysis. In particular, miR-144-5p was expressed only in the EVs of RP-treated TBI mice. Specific EVs may exist in the circulating blood of mice that escaped mortality with an ARS mitigator, and their membrane surface and endogenous molecules may be the key to the survival of mice with severe ARS.

## 1. Introduction

There is a growing concern about the risk of exposure to acute and lethal doses of ionizing radiation due to terrorist attacks and the recent threat of nuclear weapons [1,2]. In the event of a radiological or nuclear public health emergency, several thousands of people suffer severe injuries from radiation exposure, which can lead to immunosuppression and infection, bleeding, major morbidities, and even death [3,4]. Thus, there is a grave need for therapeutic strategies to ameliorate radiation-induced normal tissue toxicity and save the lives of survivors [5]. Although efforts to develop radiation medical countermeasures against acute radiation syndrome (ARS) have been underway for more than 60 years, only a limited number of effective and safe countermeasures for the exposure to lethal doses of ionizing radiation were approved by the United States Food and Drug Administration (FDA) [6,7,8,9,10]. Among these efforts, studies sponsored by the National Institute of Allergy and Infectious Diseases demonstrated that the administration of Nplate from Amgen (Romiplostim; RP), originally licensed in 2008 for the treatment of immune thrombocytopenia, increased platelet counts and improved survival in preclinical models of lethal radiation exposure [11,12]. We are also the first in the world to report the efficacy of RP as an ARS mitigator, and we have obtained many findings (e.g., enhanced DNA damage repair and antioxidant stress-responsive transcriptional factor, increased megakaryocyte hematopoiesis and mesenchymal stromal/stem cells (MSCs) in mouse hematopoietic tissues, and the suppression of vascular endothelial damage), in addition to dramatically improved survival rate of lethal total-body ionizing radiation (TBI)-exposed mice [13,14,15,16,17,18]. Although it was recently approved by the FDA as a medical countermeasure against radiation for hematopoietic ARS based on a series of successful, experimental, animal-based drug efficacy studies conducted under the Animal Rule, the detailed action mechanism of the radio-mitigative and life-saving effects of RP, which has a blood half-life of only several tens of hours, remains unclear.

Extracellular vesicles (EVs), such as exosomes, microvesicles, and apoptotic bodies, including various messenger RNA, microRNA (miRNA or miR), and proteins, are released from cells to the extracellular space. These vesicles and their contents can be taken up by other proximal or distant cells as an intercellular communication tool, and regulate various biological phenomena, such as immune responses, inflammatory reactions, and blood coagulation reactions [19,20,21]. A relationship between radiation exposure and the associated release of EVs was recently suggested. Acharya et al. reported that serum miRNAs can serve as functional dosimeters of radiation and early indicators of survival after radiation-induced hematopoietic injury [22]. Likewise, Xu et al. showed that EV-mediated miRNA transfer plays an important role in radiation-induced bystander effects, which provides new insights into the functions of miRNAs and the cellular communication between directly irradiated cells and non-irradiated cells [23]. On the other hand, MSC-derived EVs rescue mouse marrow hematopoietic cells from radiation damage and provide long-term survival of mice with ARS [24,25,26,27,28]. Thus, the efficacy of RP as an ARS mitigator is not thought to be limited to the repair of cellular damage or platelet production in radiation-exposed individuals, and it is suggested that the presence of EVs and their contents reflect the damage mitigative information to other damaged tissues/cells. Through search and identification, in addition to being able to identify the detailed mechanisms that rescue lethal radiation-exposed individuals, we expect to establish a new method for regenerating biological stem cells using EVs.

In the present study, as part of research aiming to elucidate the mechanism of the radio-mitigative effect of the domestically approved drug RP, EVs were collected from the sera of mice that overcame severe ARS and avoided mortality following the administration of RP to perform a basic study on the radio-mitigative and life-saving effects of EVs on mice acutely exposed to myelosuppressive doses of ionizing radiation.

## 2. Materials and Methods

### 2.1. Ethical Statement and Experimental Design

The Institutional Review Board Statement, approval number, and date of approval are listed in the “Institutional Review Board Statement” section. All efforts were made to minimize the number of animals used and their suffering in this study. All mice were housed in standard cages in a conventional clean room at an ambient temperature of 23 °C with 50% relative humidity, and a 12 h light/dark cycle. The mice had ad libitum access to sterilized standard laboratory mouse chow and drinking water. The number of mice used in each experiment is indicated in the corresponding figure legends.

### 2.2. In Vivo TBI with X-rays

Seven-week-old female C57BL/6JJcl mice were delivered from the breeding facilities of CLEA Japan, Inc. (Tokyo, Japan). After a week of acclimatization, 8-week-old mice were subjected to lethal TBI with 6.5 Gy of X-rays (160 kV, 3 mA, 1.0 mm aluminum filters) at a dose rate of 0.622 Gy/min using an MX-160Labo (MediXtec, Chiba, Japan) with a distance of 300 mm between the focus and the target.

### 2.3. Administration of ARS Protective/Mitigative Agents

The thrombopoietin receptor agonist RP (Romiplate^®^, Kyowa Kirin, Tokyo, Japan), as an ARS mitigator, was intraperitoneally administered once daily for 3 consecutive days to irradiated mice, starting immediately after TBI (within 2 h, 24 h, and 48 h post-TBI). The applied dose of RP was 50 µg/kg of body weight/day prepared with Normal Saline Solution (NSS; Otsuka Pharmaceutical, Tokyo, Japan) as the vehicle [13,14,15,16,17,18]. In addition to mice that received both TBI and RP (TBI + RP group), mice treated with TBI and NSS (TBI + NSS group), mice treated with RP only (0 Gy + RP group), and those treated with NSS only (0 Gy + NSS group) were prepared in this study. Furthermore, mice were also treated with Amifostine (150 mg/kg, intraperitoneal injection (AMF; WR2721, Cayman Chemical, Ann Arbor, MI, USA)) 0.5 h before TBI, as an ARS protector (AMF + TBI group), which is known to prolong survival in mice and humans by scavenging radicals produced by the indirect effects of TBI [29].

### 2.4. Serum Collection

On the 7th, 14th, 21st, and 28th days after TBI, blood was harvested from the heart (without thoracotomy) of anesthetized mice using isoflurane inhalation solution (VTRS; Viatris, Tokyo, Japan), and placed at room temperature for at least 30 min to allow blood-clotting. The serum was centrifuged at 1200× *g* for 20 min, twice, and the supernatant was then suspended in Tris-buffered saline (TBS) containing 1% EV-Save extracellular vesicle blocking reagent (FUJIFILM Wako Pure Chemicals, Osaka, Japan) as a sample for EV purification.

### 2.5. EV Purification

EVs were purified using a MagCapture Exosome Isolation Kit PS Ver.2 (FUJIFILM Wako Pure Chemicals), which adopts a novel affinity purification method using magnetic beads and phosphatidylserine (PS)-binding protein, which can easily isolate intact EVs with PS on the membrane surface with high purity and efficiency, according to the manufacturer’s instructions. In brief, Biotin-labeled Exosome Capture was immobilized on Biotin Capture magnetic beads using a magnetic stand and reacted with the sample for 1.5 h using a microtube rotator (AS ONE, Osaka, Japan). After washing three times with wash buffer containing 0.2% exosome binding enhancer, EVs were eluted with exosome elution buffer containing 1% EV-Save extracellular vesicle blocking reagent. Filtration treatment was performed at 12,000× *g* for 4 min using Ultrafree-MC 0.45 μm (Merck Millipore, Darmstadt, Germany) since there was a possibility that the pooled eluant contained a small number of magnetic beads, and EVs were kept in cold storage until the next step.

### 2.6. Estimation of the Total Amount of Recovered EVs

Using a Bicinchoninic Acid Protein Assay Kit (FUJIFILM Wako Pure Chemicals), the extract and reagents were reacted at 37 °C for 30 min, and the absorbance at 562 nm was measured using an iMark microplate reader (Bio-Rad, Tokyo, Japan). We determined the total protein content in EVs. The standard curve was prepared using serial dilutions of 2 mg/mL albumin solution from bovine serum. The estimated amount of EVs was evaluated based on the acetyl-CoA acetylcholinesterase activity, which was determined using a FluoroCet Exosome Quantitation Kit (System Biosciences, Palo Alto, CA, USA) according to the manufacturer’s instructions. In brief, after adjusting the protein concentration to 10 ng/μL, it was allowed to react with lysis buffer on ice for 30 min and mix with 0.5 M acetylcholine chloride at room temperature for 20 min in the dark. Fluorescence (measurement wavelength: excitation 530 nm/emission 585 nm) was measured using a Mithras LB940 plate reader (Berthold, Tokyo, Japan). The standard curve was created using serially diluted FluoroCet standard, which was supplied within the kit.

### 2.7. Detection of Membrane Surface Proteins of EVs

Membrane surface proteins of EVs were detected using a PS Capture Exosome Flow Cytometry Kit (FUJIFILM Wako Pure Chemicals) according to the manufacturer’s instructions. In brief, EVs added with exosome capture beads and 1% exosome binding enhancer were allowed to react at room temperature for 1 h using a magnetic stand. After washing twice with wash buffer containing 1% exosome binding enhancer, fluorescence-labeled isotype control antibodies or exosome surface antigen antibodies were allowed to react at room temperature for 1 h. Washing was performed three times with wash buffer containing 1% exosome binding enhancer and then analyzed by flow cytometry (FC500; Beckman Coulter, Fullerton, CA, USA). The fluorescence antibodies were as follows: fluorescein isothiocyanate (FITC)-labeled rat IgG2a kappa, phycoerythrin (PE)-labeled rat IgG2a kappa, and PE-cyanine5 (PC5)-labeled rat IgG2a kappa were used for isotype control antibodies (Invitrogen, Tokyo, Japan). FITC-labeled CD9 monoclonal antibody, biotin-labeled CD81 monoclonal antibody (Invitrogen), PE-labeled anti-mouse CD63 antibody (BioLegend, Tokyo, Japan), and PC5-labeled Streptavidin antibody (System Biosciences) were used for fluorescence-labeled exosome surface antigen antibodies.

### 2.8. Administration of EVs to Mice with Severe ARS

Eight-week-old female C57BL/6JJcl mice were subjected to lethal TBI with 6.5 Gy of X-rays under the same conditions as in the above section, and then the EVs were intraperitoneally administered once daily for three days. The applied dose of EVs was approximately 10^6^ to 10^7^ particles/100 μL prepared with NSS as the vehicle. Survival was monitored for up to 30 days.

### 2.9. RNA Extraction from Purified EVs

RNA, especially miRNA, was extracted from purified EVs using a microRNA Extractor SP Kit (FUJIFILM Wako Pure Chemicals) according to the manufacturer’s instructions. Approximate RNA concentrations were assessed using a NanoDrop spectrophotometer (NanoDrop Technologies, Wilmington, DE, USA). In addition, the peaks and concentrations of small RNAs were confirmed using Agilent 2100 Bioanalyzer and an Agilent RNA 6000 Pico kit (Agilent Technologies, Santa Clara, CA, USA) according to the manufacturer’s instructions.

### 2.10. Microarray Analysis

Cyanine 3 (Cy3)-labeled miRNA was synthesized from 30 ng small RNA of purified EVs using a miRNA Complete Reagent and Hyb kit (Agilent Technologies). A SurePrint G3 mouse miRNA microarray slide (8 × 60 K, Ver.21.0) was hybridized with the Cy3-labelled miRNA in a hybridization solution prepared with a Gene Expression Hybridization Kit according to the manufacturer’s instructions. Cy3 fluorescence signal images on the microarray slide were obtained by a SureScan microarray scanner and processed using the Feature Extraction software program (version 10.7) based on the instructions of Agilent Technologies. The expression data were processed using the GeneSpring GX14.9 software program (Available from 1 November 2021 to 31 January 2022.) to normalize to a 90% percentile shift of all values on the respective microarrays, followed by normalization of the median expression level of all samples. After filtering entities by expression level and quality information, the expression profiles of miRNAs were compared based on the fold-change of the signal values of respective genes and Student’s *t*-test with a cut-off *p* value of 0.05. miRNAs that showed a >2.0-fold increase or decrease were selected. Results are visualized with the help of a volcano plot, scatter plot, and heat map with dendrograms. The volcano and scatter plots were used to enable the visualization of the relationship between fold change and statistical significance, which showed that miRNA expression levels change as each plot moved away from the center; color gradation from blue to red indicates low to high expression levels, respectively. The heat maps show color-coded expression levels; color gradation from green to red indicates low to high expression levels, respectively. Sample trees were drawn horizontally, and gene trees were drawn vertically.

### 2.11. Reverse Transcription-Quantitative Polymerase Chain Reaction (RT-qPCR)

The expression levels of miRNAs from purified EVs were analyzed using cDNAs that were synthesized from miRNAs using a TaqMan MicroRNA Reverse Transcription Kit (Applied Biosystem) and the prescribed 5 × RT primer (Assay ID: 464811_mat and 470724_mat) according to the manufacturer’s instructions. Subsequently, qPCR for miRNAs was performed using TaqMan Fast Advanced Master Mix, 20 × TM probe, and a StepOne Plus real-time PCR system (Thermo Fisher Scientific, Boston, MA, USA) under the following conditions: pre-incubation at 50 °C for 2 min, denaturation at 95 °C for 20 s, and incubation with 40 thermal cycles consisting of 95 °C for 1 s and 60 °C for 20 s. U6 small nuclear RNA (snRNA) was used as an internal control (Assay ID: 001973). Expression levels were determined using the comparative Ct method. The information on the action sequences of miRNAs was obtained from the miRBase database (Release 22.1, https://www.mirbase.org/ (accessed on 12 Octorber 2021), Manchester, UK).

### 2.12. Functional Annotation of miRNA

DNA Intelligent Analysis (DIANA)-miRPath v3.0 (http://www.microrna.gr/miRPathv3 accessed on 1 November 2022) is an online software suite dedicated to conducting functional and pathway enrichment analyses for miRNAs. In our study, the DIANA-microT-CDS target prediction algorithm was employed to predict miRNA targets. This was combined with a Kyoto Encyclopedia of Genes and Genomes (KEGG) analysis, which can provide specific pathways and link genomic information with higher-order functional information, and a gene ontology (GO) analysis that can annotate the functions of genes using terms from a dynamic, controlled vocabulary, which contains three aspects of biology, including biological processes, cellular components, and molecular functions. A target prediction threshold of 0.8, with a *p* value of 0.05, and false discovery rate correction were applied.

### 2.13. Statistical Analysis

Significant differences between the two groups were analyzed using Student’s *t*-test, while a Kaplan–Meier analysis followed by Mantel–Cox (log-rank) test was used to analyze survival, respectively. The levels of significance were calculated using the Excel 2016 software program (Microsoft, Redmond, WA, USA) with the Statcel3 add-on (OMS, Saitama) and OriginPro 2018b (Origin Lab, Northampton, MA, USA). *p* values of <0.05 were considered to indicate statistical significance.

## 3. Results

### 3.1. EVs Collected from Mice That Overcame ARS with RP Treatment Had Life-Saving Effects in Mice with Severe ARS

**Figure 1:**
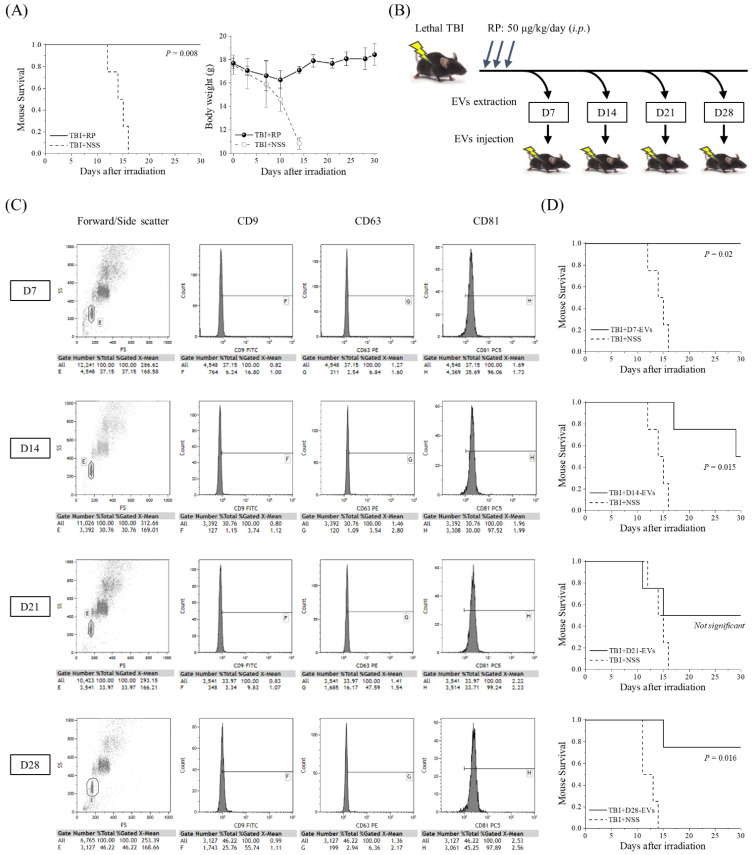
Life-saving effect of EVs collected from mice that overcame ARS with the administration of RP. (**A**) A Kaplan–Meier plot for the survival of 6.5 Gy X-irradiated C57BL/6JJcl female mice treated with RP as an ARS mitigator (TBI + RP, *n* = 4) or NSS as the vehicle (TBI + NSS, *n* = 4). The statistical significance of the difference was determined by a log-rank test. *p* values of <0.05 were considered to indicate statistical significance. Body weight changes are shown until day 30 after TBI. The data are expressed as the mean ± standard deviation. (**B**) Serum was harvested from TBI + RP mice on the 7th, 14th, 21st, and 28th days after TBI, and approximately 10^6^ to 10^7^ EVs were administered once daily, three times, to mice subjected to TBI. Survival was monitored for up to 30 days. (**C**) The detection of EV membrane surface proteins, such as CD9, CD63, and CD81, was performed by flow cytometry. Representative dot plots of forward/side scatter and histograms of the expression of each membrane surface protein are shown. The expression levels of each isotype control antibody were used as a reference. (**D**) Kaplan–Meier plots for the survival of TBI mice treated with TBI + RP EVs on the 7th, 14th, 21st, and 28th days (TBI + D7-EVs, TBI + D14-EVs, TBI + D21-EVs, and TBI + D28-EVs, each *n* = 4) or NSS as the vehicle (TBI + NSS, *n* = 4). The statistical significance of differences, in comparison to TBI + NSS mice, was determined by a log-rank test. *p* values of <0.05 were considered to indicate statistical significance.

The thrombopoietin receptor agonist RP was recently approved by the FDA to improve survival in patients acutely exposed to myelosuppressive doses of radiation [11,12,13,14,15,16,17,18]. To confirm the radio-mitigative and life-saving effects of RP in our mouse model of severe ARS caused by lethal TBI, we monitored the changes in body weight and the level of animal survival over a period of 30 days (Figure 1A). RP completely suppressed the TBI-induced lethality and significantly increased the survival rate (*p* = 0.008), whereas in the TBI + NSS mice, it gradually decreased from day 12 after TBI and reached 0% on day 16. Body weight gradually increased in the TBI + RP mice from day 10, whereas it decreased in the TBI + NSS mice, starting immediately after TBI. Since the radio-mitigative effect of RP was confirmed, the serum was harvested from the TBI + RP mice as a sample for EV purification on the 7th, 14th, 21st, and 28th days after TBI. EVs were characterized by flow cytometry and then approximately 10^6^ to 10^7^ EVs were administered once daily for three days to mice with severe ARS induced by TBI to evaluate the life-saving effects of their EVs (Figure 1B). Fluorescence-labeled antibodies against CD9, CD63, and CD81 proteins, which are members of the four-transmembrane protein highly enriched in EVs, were used to analyze the expression of the membrane surface proteins of the extracted EVs [30]. Fractions in which exosome capture beads were not aggregated from the plot diagram composed of forward scattered (FS) and side scattered (SS) light were gated, and then the fluorescence intensity contained inside the gate was compared. These expression levels were confirmed in a preliminary experiment using D0-EVs collected from 0 Gy + NSS mice (CD9 positive rate 98%, CD63 positive rate 42%, and CD81 positive rate 99% based on the expression levels of isotype control). This method could identify our isolated samples as EVs. EVs on each collection day consistently expressed a high positive rate of CD81 at >96%, but the positive rate of CD9 and CD63 varied with radiation exposure and time course [31] (Figure 1C). All the TBI + NSS mice died until day 16, whereas in the TBI + D7-EVs, TBI + D14-EVs, and TBI + D28-EVs, the 30-day survival rate of mice with severe ARS was 100% (*p* = 0.02), 50% (*p* = 0.015), and 75% (*p* = 0.016), respectively (Figure 1D). In the TBI + D21-EVs mice, the 30-day survival rate improved by 50%, but no statistically significant difference was observed (*p* = 0.287). These results demonstrated that the EVs collected from the sera of mice that overcame ARS and avoided mortality due to the administration of RP showed life-saving effects in mice with severe ARS.

### 3.2. EVs That Contribute to the Survival of Mice with ARS Were Present in the Blood of Mice That Overcame ARS

**Figure 2:**
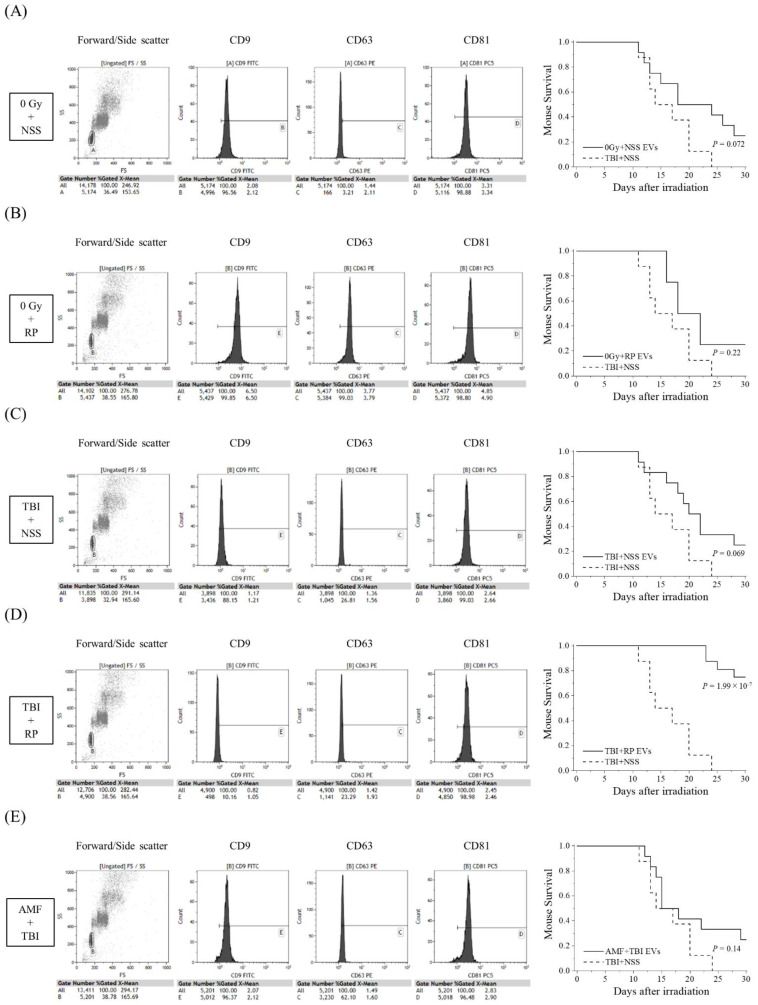
Comparative study of life-saving effects of each EV on TBI mice. The detection of EV membrane surface proteins, such as CD9, CD63, and CD81, and Kaplan–Meier plots of the survival of TBI mice treated with EVs collected from (**A**) 0 Gy + NSS mice (0 Gy + NSS EVs, *n* = 12); (**B**) 0 Gy + RP mice (0 Gy + RP EVs, *n* = 12); (**C**) TBI + NSS mice (TBI + NSS EVs, *n* = 12); (**D**) TBI + RP mice (TBI + RP EVs, *n* = 16); and (**E**) AMF + TBI mice (AMF + TBI EVs, *n* = 12) on the 7th day or NSS as vehicle (TBI + NSS, *n* = 8). The statistical significance of differences, in comparison to TBI + NSS mice, was determined by a log-rank test. *p* values of <0.05 were considered to indicate statistical significance.

Since D7-EVs collected from the TBI + RP mice had a higher life-saving effect on mice with severe ARS in comparison to EVs collected on other days, and because EVs could even be collected in the TBI + NSS mice on day 7 after TBI, an additional study of the survival rate was performed focusing on this recovery date. In addition to the TBI + RP mice, 0 Gy + NSS mice, 0 Gy + RP mice, TBI + NSS mice, and AMF + TBI mice were prepared for the collection of EVs on day 7 after TBI to compare the life-saving effects of the EVs of each group. EVs were characterized by flow cytometry and then approximately 10^6^ to 10^7^ EVs were administered once daily, three times, to mice with severe ARS induced by TBI (Figure 2). EVs collected from the 0 Gy + NSS mice, 0 Gy + RP mice, TBI + NSS mice, and AMF + TBI mice consistently expressed a high positive rate of CD81 (>96%), similar to TBI + RP EVs (Figure 1C); however, the positive rates of CD9 and CD63 varied with radiation exposure and ARS protector/mitigator treatment. In the TBI + NSS mice, it gradually decreased from day 12 after TBI and reached 0% on day 24, whereas the 30-day survival rate of mice with severe ARS treated with TBI + RP EVs improved by 75% (*p* = 1.99 × 10^−7^, Figure 2D). In mice with severe ARS treated with 0 Gy + NSS EVs (Figure 2A), 0 Gy + RP EVs (Figure 2B), and TBI + NSS EVs (Figure 2C), the 30-day survival rate improved to 25%; however, the difference did not reach statistical significance (*p* = 0.072, *p* = 0.22, and *p* = 0.069, respectively). Furthermore, EVs collected from the TBI mice pretreated with AMF (a radioprotector) were unable to confer a significant survival improvement in mice with ARS (30-day survival rate 25%, *p* = 0.14, Figure 2E). These results suggested that EVs may exist in the circulating blood of mice that escaped mortality with ARS mitigator treatment, contributing to the reduction in radiation damage and improving survival of mice with severe ARS.

### 3.3. Specific miRNA Contained in EVs That Have Life-Saving Effects in Mice with Severe ARS

**Figure 3:**
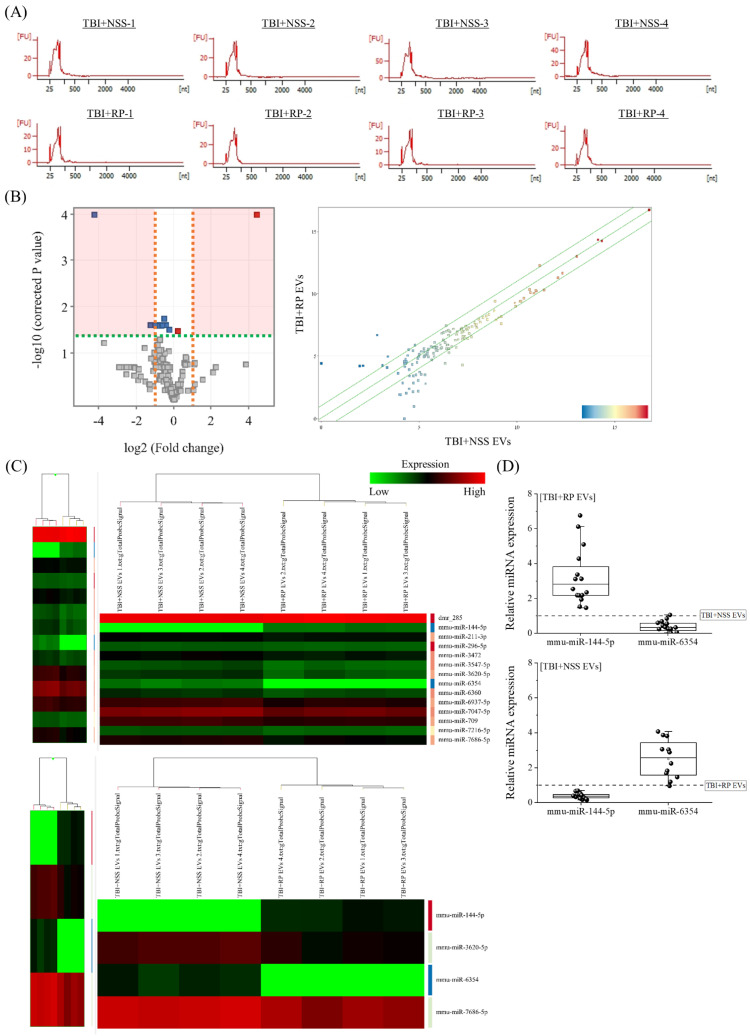
Identification of differentially expressed miRNAs in EVs that exhibited life-saving effects and EVs that did not exhibit life-saving effects. (**A**) Detection of small RNA peaks in TBI + RP EVs and TBI + NSS EVs (each *n* = 4) using an Agilent 2100 Bioanalyzer. Peaks were observed in the low-molecular-weight RNA region but not in the high-molecular-weight RNA region, and EV-derived RNA was recovered without cell-derived RNA contamination. Expression analysis of miRNAs in TBI + RP EVs and TBI + NSS EVs was conducted using an Agilent mouse miRNA microarray. The results were visualized with the help of (**B**) a volcano plot and a scatter plot. In the volcano plot, the vertical lines correspond to a 2.0-fold increase or decrease in an expression level, while the horizontal line represents a *p* value of 0.05. Gray points in the plot represent miRNAs with no statistical differences, and red/blue points represent significantly upregulated/downregulated miRNAs. A scatter plot was constructed to evaluate the distribution of data in the two different EVs. (**C**) A heat map with dendrograms showed the variation in 14 responsive miRNAs out of 145 miRNAs, which were identified by Student’s *t*-test with a cut-off *p* value of 0.05, and 4 responsive miRNAs that showed a >2.0-fold change, in addition to the above *p* value using the GeneSpring GX14.9 software program. (**D**) The expression of miR-144-5p and miR-6354 in TBI + RP EVs and TBI + NSS EVs (each *n* = 16), as evaluated by RT-qPCR using comparative Ct methods and normalized using the values of other EVs set at 1.0 (dotted line). Box plots show the 25%, 50%, and 75% percentiles; whiskers show maximum and minimum values. U6 snRNA was used as an internal control. The statistical significance of differences was determined by Student’s *t*-test. *p* values of <0.05 were considered to indicate statistical significance.

Next, we focused on miRNA to clarify the endogenous molecules in EVs that contribute to life-saving effects in mice with severe ARS. RNA, especially miRNA, was extracted from the TBI + RP EVs, which showed life-saving effects in mice with ARS (Figure 2D), and the TBI + NSS EVs, which did not show life-saving effects (Figure 2C), for comparison. The peaks of 25–200 nucleotides in the low-molecular-weight RNA region were detected, and 18S ribosomal RNA (1874 nucleotides)/28S ribosomal RNA (4718 nucleotides) in the high-molecular-weight RNA region were not detected by an Agilent 2100 Bioanalyzer, indicating a high level of extracellular small RNAs in purified EVs of each mouse (Figure 3A). To confirm the differences in endogenous molecules, especially the miRNA expression in both EVs, we performed an Agilent mouse miRNA microarray assay. A total of 145 high-intensity signals were filtered after the exclusion of low-intensity signals (from a total of 1902). A principal component analysis and scatter plot (Figure 3B) were used to evaluate the data distribution in each EV to guarantee the accuracy and reliability of data, and this evaluation showed a relatively good representativeness of the specimens applied in this study. miRNAs that were significantly differentially expressed were identified by a volcano plot analysis, and we filtered a total of 14 (2 upregulated and 12 downregulated) miRNAs (Figure 3B). A hierarchical cluster analysis demonstrated that miRNAs in the TBI + RP EVs could be distinguished from that in the TBI + NSS EVs based on their miRNA expression patterns, which showed the variation in 14 responsive miRNAs identified by Student’s t-test with a cut-off *p* value of 0.05, and 4 responsive miRNAs, identified based on more than 2.0-fold changes in addition to the above *p* value using the GeneSpring GX14.9 software program (Figure 3C). Fourteen miRNAs were listed by systematic name, accession number, and active sequence based on the miRBase database (Table 1). We then chose miR-144-5p and miR-6354 as candidate endogenous miRNAs that might be involved in life-saving effects in mice with severe ARS since miR-144-5p was only included in the TBI + RP EVs (not in TBI + NSS EVs) and miR-6354 was only included in the TBI + NSS EVs (not in TBI + RP EVs) when quantifying the hierarchical clustering data using a software program, and selected miRNAs underwent further validation by RT-qPCR (Figure 3D). As a result, the expression of miR-144-5p and miR-6354 in the TBI + RP EVs showed 2.83-fold (*p* = 1.4 × 10^−6^) and 0.33-fold changes (*p* = 6.3 × 10^−7^), respectively, in comparison to their expression in the TBI + NSS EVs, while the expression of both miRNAs in the TBI + NSS EVs showed 0.36-fold (*p* = 1.1 × 10^−10^) and 2.56-fold changes (*p* = 5.9 × 10^−4^), respectively, in comparison to their expression in the TBI + RP EVs. It was revealed that EVs that showed life-saving effects in mice with severe ARS contain miR-144-5p but not miR-6354, and EVs that did not show life-saving effects contained miR-6354 but not miR-144-5p, suggesting that these miRNAs may contribute to the reduction in radiation damage and improvement of survival in mice with severe ARS.

### 3.4. Functional Annotations of Differentially Expressed miRNAs in EVs

**Figure 4:**
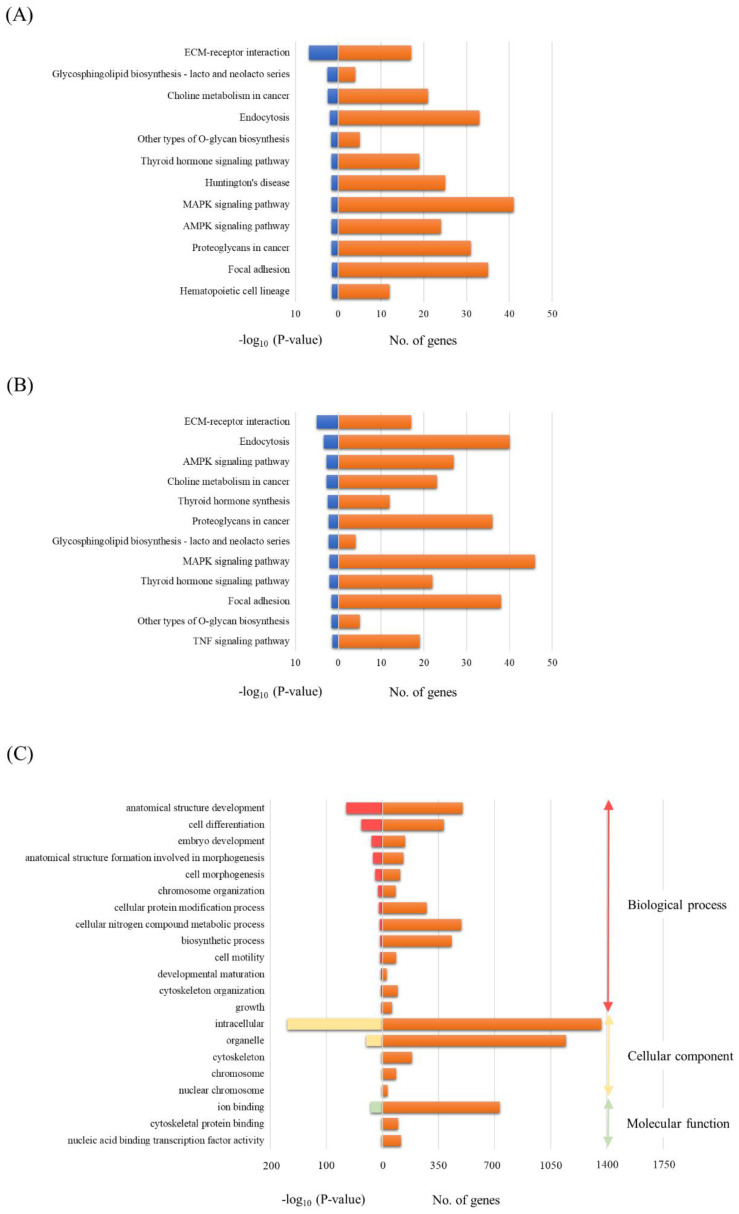
The bioinformatics analysis of miRNAs in EVs that exhibit life-saving effects and EVs that did not exhibit life-saving effects. A KEGG analysis was used to identify all the potential pathways corresponding to miRNAs in (**A**) TBI + RP EVs and (**B**) TBI + NSS EVs. (**C**) A GO analysis was used to investigate the pathways associated with biological processes, cellular components, and specific molecular functions corresponding to the target genes of miRNAs in TBI + RP EVs. These analyses are identified using a web-based DIANA-miRPath v3.0, as demonstrated by the number of genes and a −log_10_ (*p*-value) of >1.3.

To investigate the potential biological functions of miRNAs differentially expressed in both the TBI + RP EVs and TBI + NSS EVs, the assessment of miRNA regulatory roles and the identification of controlled pathways was performed using the DIANA-miRPath v3.0 software program [32]. The analysis of the 13 differentially expressed miRNAs excluding miR-6354 in the TBI + RP EVs based on microarray data revealed that these miRNAs targeted 12 different KEGG pathways, and the 13 differentially expressed miRNAs (excluding miR-144-5p derived from the TBI + NSS EVs) also targeted 12 different pathways with a high degree of significance (*p* < 0.05). Both EVs had common pathways, including extracellular matrix (ECM)-receptor interaction; glycosphingolipid biosynthesis-lacto and neolacto series; choline metabolism in cancer; endocytosis; other types of O-glycan biosynthesis; thyroid hormone signaling pathway; mitogen-activated protein kinase (MAPK) signaling pathway; adenosine monophosphate-activated protein kinase (AMPK) signaling pathway; proteoglycan in cancer; and focal adhesion (Figure 4A,B). On the other hand, the pathway related to Huntington’s disease and hematopoietic cell lineage in the TBI + RP EVs (Figure 4A) and the thyroid hormone synthesis and tumor necrosis factor (TNF) signaling pathway in the TBI + NSS Evs (Figure 4B) were nominated as characteristic pathways in each EV. Twelve candidate genes were specifically targeted in the pathway related to the hematopoietic cell lineage as follows: glycoprotein 5 (*Gp5*); interleukin 7 (*Il7*); integrin alpha 5 (*Itga5*); interleukin 2 receptor alpha chain (*Il2ra*); CD38 antigen (*Cd38*); kit ligand (*Kitl*); CD33 antigen (*Cd33*); membrane-spanning 4-domains subfamily A member 1 (*Ms4a1*); integrin alpha 4 (*Itga4*); colony-stimulating factor 1 (*Csf1*); CD44 antigen (*Cd44*); and transferrin receptor (*Tfrc*). A more detailed target prediction analysis of the 13 miRNAs, excluding miR-6354 in the TBI + RP EVs, was performed by applying a GO enrichment analysis (Figure 4C). The GO pathway annotates different genes and gene products to certain gross biological terms, such as biological process and subcellular localization. The top GO processes, predicted to be influenced by these 13 miRNAs, were development, cell differentiation, cell morphogenesis, metabolic and biosynthetic process, cell motility, and growth. This analysis also showed that all miRNAs within these processes were located in the following cellular components: intracellular, organelle, cytoskeleton, and nuclear chromosome. The important signaling pathways targeted by the miRNAs contained in EVs that have life-saving effects in mice with severe ARS may be related to the hematopoiesis systems in this approach.

## 4. Discussion

In a high-dose radiation-exposed individual, top priority is given to treatments intended to reconstitute and restore hematopoiesis. In many patients who experience radiological accidents, drug therapy is the most suitable initial treatment, and therefore, a stable supply and regular stockpile of approved pharmaceutical drugs are desired. Our previous studies showed that the thrombopoietin receptor agonist RP, a drug used for the treatment of idiopathic thrombocytopenic purpura, completely rescued mice exposed to lethal TBI [13], and the radio-mitigative effects of RP were also reported by other groups [11,12]. However, the mechanism of action of RP cannot be explained by signal transduction via the thrombopoietin receptor alone, and significant efforts were made to understand the mechanisms for achieving this life-saving effect [15,16,17,18]. In the present study, to further elucidate the radio-mitigative effect mechanism of the domestically approved drug RP, we focused on EVs that were collected from the sera of mice that overcame severe ARS and avoided mortality with the administration of RP, and performed a basic study on the radio-mitigative and life-saving effects of their EVs on mice acutely exposed to myelosuppressive doses of ionizing radiation.

As a result of this study, the 30-day survival rate of lethal TBI mice drastically improved by 50–100% with the administration of EVs in the sera collected weekly from mice in which radiation damage was alleviated and mortality was avoided by the administration of RP (Figure 1), suggesting the possibility that EVs may reduce radiation damage and rescue mice with severe ARS. Furthermore, the life-saving effect was observed only in EVs recovered from RP-treated TBI mice (30-day survival rate 75%, *p* = 1.99 × 10^−7^), but not in the other groups (0 Gy + NSS, 0 Gy + RP, and TBI + NSS mice) (Figure 2). In particular, although AMF could protect treated animals from high doses of ionizing radiation by scavenging free radicals [29], EVs contained in the blood circulation of AMF + TBI mice had no life-saving effect, which may be due to the difference in the mechanism of action of RP on mice with ARS (Figure 2E). Our research team used a mouse model of severe ARS and reported that a single administration of RP produced a 100% survival rate in C57BL/6J mice exposed to a lethal TBI and that all irradiated mice survived for more than 30 days with both 3- and 5-day consecutive administrations [14]. Along with the recovery and promotion of the hematopoietic function in the bone marrow, lungs, and spleen, a rapid increase was observed in the MSCs present in mesenchymal tissue (i.e., mesoderm-derived connective tissue) with a suppressive effect on vascular endothelial damage. The functions of MSCs include homing to damaged sites and differentiation/replenishment, the role as a niche that creates a special environment that controls the differentiation and proliferation of progenitor cells, and the EVs that encapsulate proteins and nucleic acids supply to surrounding damaged cells. Wen et al. reported the intravenous administration of human MSC-derived exosomes (4 × 10^9^ particles) to C57BL/6 mice exposed to 5 Gy γ-rays (6, 24, and 72 h after irradiation) suppressed damage to the bone marrow hematopoietic cells [24], while Schoefinius et al. improved the survival rate of radiation-exposed individuals to 60% without hematopoietic stem cell transplantation by the intravenous administration of exosomes derived from mouse MSCs to the C57BL/6 mice exposed to 9.5 Gy γ-rays [25]. In addition, Piryani et al. demonstrated that the intravenous administration of mouse vascular endothelial cell-derived exosomes (1.9–4.9 × 10^9^ particles) to the C57BL/6 mice with whole-body exposure to 8 Gy γ-rays (24, 48, 72 h, and 96 h after irradiation) improved the survival rate of radiation-exposed individuals by up to 50% [33]. It is suggested that EVs reflect the properties of the cells from which they are derived, and that delivered molecules change the phenotypes of cells that receive EVs, contributing to the control of various biological phenomena, such as the hematopoietic function and immune regulation [34]. Furthermore, Zhang et al. reported that the injection of 100 μL serum derived from non-radiated mice to whole-body radiation-exposed mice through the tail vein once every other day for up to 14 days improved the blood circulation environment of the exposed individuals, and the survival percentage of the radiated mice increased to 50% with serum injection, indicating that the injection of serum from non-radiated mice markedly protects the radiated mice against hematopoietic system injury [27]. Since they suggested that EVs contained in the serum are important active components that contribute to radio-mitigative and life-saving effects, in this study, the serum EVs recovered from mice that overcame severe ARS and avoided mortality with the administration of RP-contained endogenous molecules and biological information that controlled injury reduction/healing, which may have a life-saving effect in individuals exposed to lethal TBI.

We then focused on miRNAs to clarify the endogenous molecules in EVs recovered from RP-treated TBI mice that contribute to the life-saving effects in mice with severe ARS, which showed variation in four responsive miRNAs, namely, miR-144-5p, miR-3620-5p, miR-6354, and miR-7686-5p that showed more than 2.0-fold changes in addition to Student’s *t*-test with a cut-off *p* value of 0.05 (Figure 3). We found that EVs that had life-saving effects in mice with severe ARS contained miR-144-5p, but not miR-6354, and that EVs that did not show life-saving effects contained miR-6354, but not miR-144-5p, suggesting that these miRNAs may contribute to the reduction in radiation damage and the improvement of survival of mice with severe ARS. To our knowledge, this is a new finding, as few previous reports have shown an association between the radiation exposure/radio-mitigative effects and the expression of these miRNAs. Among the specific miRNAs contained in EVs that showed life-saving effects in mice with severe ARS, miR-144-5p, in particular, is known to exhibit anti-tumor activities in various cancers [35,36]. In addition, miR-144-5p is involved in many biological processes, including cell proliferation, stem cell differentiation, inflammation, and apoptosis. Zhang et al. showed that miR-144-5p silencing aggravated erythrocytosis and hyperviscosity, and also accentuated lung tissue damage and excessive accumulation of red blood cells via the erythropoietin/erythropoietin receptor in the hypobaric hypoxia-induced chronic mountain sickness model of rats [37]. Several reports also demonstrated the reduction in lipid deposition, suppression of oxidative stress, and the inflammatory response in mice with non-alcoholic fatty liver disease [38]; the enhancement of cell viability, suppression of cell apoptosis, fibrosis, and pyroptosis in a cardiomyocyte model [39]; and the restoration of angiogenesis and the nerve function [40] via miR-144-5p expression. In addition, Yang et al. provided speculation about the tissue of origin, suggesting that MSC-derived exosomal miR-144-5p modulates vascular endothelial injury [41] and improves the rat ovarian function after chemotherapy-induced ovarian failure by targeting the Akt/phosphatase and tensin homolog pathway [42], which indicates an association between increased MSCs and the expression of miR-144-5p in the TBI + RP mice. Higher miR-7686-5p targeted the Wnt/β-catenin signaling, retinoic acid receptor activation, apoptosis, signal transducer, and activator of transcription 3, and signaling of several IL families, which have been identified in the apoptosis and cell survival canonical pathways [43]. Regarding miR-3620-5p, the gene silencing of miRNA causes calcification/senescence in human aortic vascular smooth muscle cells [44] and it is a target miRNA for nuclear factor-κB, which is known to regulate a wide range of biological functions, including cell survival, proliferation, differentiation, inflammation, immunity, and tumorigenesis [45]. Unfortunately, there were no reports about the association of miR-6354 with radiation exposure or biological effects, but it is known that EV-mediated miRNA transfer plays an important role in radiation-induced bystander effects [23]. miR-6354 was specifically contained in EVs that showed no life-saving effect on mice with ARS, providing the possibility that this miR-6354 is related to the cellular communication between irradiated cells and other proximal or distant cells. Taken together, the identified miRNAs from EVs that have life-saving effects in mice with severe ARS may play important roles in cell survival, anti-apoptosis, anti-oxidative stress, anti-inflammatory responses, and the promotion of the hematopoietic system. We also think that EVs and these miRNAs from this study could be important players in the mechanisms of reducing disability and saving/prolonging life in individuals exposed to lethal TBI, and their role in this process is worthy of further elucidation.

We are currently conducting a comprehensive analysis of proteins endogenous to EVs that were found to have radiation damage reduction/life-saving effects, the identification of the organ of origin of EVs, and demonstrate direct life-saving and radio-mitigative effects by synthesizing the identified endogenous molecules and administering them to mice with severe ARS. However, since there are no data on the expression of membrane surface proteins other than CD antigens in purified EVs, morphology evaluation under transmission electron microscopy, and granular distribution by nanoparticle tracking analysis, a more detailed evaluation of EVs is required. Furthermore, additional studies in dual gender and various mouse strains should be performed to firmly establish the mechanism of action and the validation of the effect on ARS. Medical preparations for radiation exposure accidents are now a common issue for countries with nuclear power plants. It is also expected that this study will lead to social and medical contributions, such as the alleviation of hematopoiesis in radiation-exposed individuals, enhancement of their viability, and adaptation to the alleviation of side effects such as cancer radiotherapy-associated myelosuppression.

## 5. Conclusions

EVs may exist in the circulating blood of mice that escaped mortality with an ARS mitigator, and their endogenous molecules (e.g., miR-144-5p and miR-6354) may be the key to the reduction in radiation damage and survival of mice with severe ARS.

## Figures and Tables

**Table 1:** Candidate miRNA list encapsulated in EVs showing life-saving effects in mice with ARS.

Systematic Name	Accession Number	Active Sequence
mmu-miR-144-5p	MIMAT0016988	GGAUAUCAUCAUAUACUGUAAGU
mmu-miR-211-3p	MIMAT0017059	GCAAGGACAGCAAAGGGGGGC
mmu-miR-296-5p	MIMAT0000374	AGGGCCCCCCCUCAAUCCUGU
mmu-miR-3472	MIMAT0015643	UAAUAGCCAGAAGCUGGAAGGAACC
mmu-miR-3547-5p	MIMAT0027832	GUGGGAAGAGGGGUGGGGCCCGGGA
mmu-miR-3620-5p	MIMAT0029878	CUGUGGGCUGGGCUGGGAAGCA
mmu-miR-6354	MIMAT0025097	UGCCCUGGGGAUCAGGUCUCU
mmu-miR-6360	MIMAT0025103	UAGUGUUGCUCAGGCAGCAGGA
mmu-miR-6937-5p	MIMAT0027774	UAGCUGUAAGGGCUGGGUCUGUGU
mmu-miR-7047-5p	MIMAT0027998	UGAGGGAGGAGGGCUGGGUCUGA
mmu-miR-709	MIMAT0003499	GGAGGCAGAGGCAGGAGGA
mmu-miR-7216-5p	MIMAT0028400	UGGAGAGCUGGCAGAGGACCCAGA
mmu-miR-7686-5p	MIMAT0029898	CCUUCCACUGGACCUGGGGCUGGGC

## Data Availability

The datasets used and/or analyzed during the current study are available from the corresponding authors upon reasonable request.
